# Coronary angiography–derived index of microcirculatory resistance associated with contrast-induced acute kidney injury in patients with STEMI

**DOI:** 10.3389/fcvm.2025.1541208

**Published:** 2025-05-01

**Authors:** Sifang Zhong, Jinyang Lu, Kaiyue Gong, Yixuan Wu, Zishuang Dong, Yuan Lu

**Affiliations:** ^1^ Department of Cardiology, Xuzhou Central Hospital, Xuzhou, China; ^2^ Department of Cardiology, The Affiliated Hospital of Xuzhou Medical University, Xuzhou, China

**Keywords:** cardiovascular disease, contrast-induced acute kidney injury, STEMI, coronary microvascular dysfunction, index of microcirculatory resistance

## Abstract

**Background:**

More than half of ST-segment elevation myocardial infarction (STEMI) patients have coronary microcirculatory dysfunction (CMD) after percutaneous coronary intervention (PCI). This study aimed to explore the role of CMD in the occurrence of contrast-induced acute kidney injury (CI-AKI) in patients with STEMI.

**Methods:**

This was a single-centre retrospective clinical observational study. Coronary angiography–derived index of microcirculatory resistance (caIMR) was measured and used to assess CMD. Regression analysis was used to identify risk factors for CI-AKI. Restricted cubic splines (RCS) was employed to examine the dose-response relationship between caIMR and CI-AKI. The predictive accuracy of the models was assessed with net reclassification index (NRI), and integrated discrimination improvement (IDI).

**Results:**

This study included 745 patients, the incidence of CI-AKI was 10.6% (79/745). Multivariate logistic regression identified caIMR (OR = 1.072, 95% CI: 1.051–1.094) as an independent predictor of CI-AKI. RCS analysis indicated a linear dose-response relationship between caIMR and CI-AKI. Receiver operating characteristic (ROC) analysis demonstrated that the areas under the curve for caIMR was 0.725, the optimal cutoff value was 25.95 U. Integration of caIMR could significantly improve the risk model for CI-AKI in STEMI patients (NRI = 0.721, IDI = 0.102, *P* < 0.001).

**Conclusions:**

Elevated caIMR is an independent risk factor for the development of CI-AKI after PCI in STEMI patients. Integrating caIMR significantly improves the risk model for CI-AKI.

## Introduction

With the acceleration of population aging, the burden of cardiovascular diseases is also increasing. Among them, ST-segment elevation myocardial infarction (STEMI) poses a significant challenge to human health (1). Percutaneous coronary intervention (PCI) is currently the main treatment for STEMI patients, but 15%–35% of patients develop contrast-induced acute kidney injury (CI-AKI) after PCI (2). Evidence suggests that STEMI is one of the key factors contributing to the increased incidence of CI-AKI and the need for dialysis, which may be related to the hemodynamic instability, neuroendocrine activation, and intense inflammatory response in STEMI patients (3, 4). Although CI-AKI is associated with poor prognosis, there is currently a lack of specific clinical drugs and interventions for preventing CI-AKI (5). Therefore, early identification of high-risk populations and active prevention of CI-AKI is crucial.

Although PCI successfully restores coronary epicardial blood flow, more than half of STEMI patients still experience coronary microcirculatory dysfunction (CMD) (6, 7). The index of microcirculatory resistance (IMR) is the “gold standard” for quantitatively measuring coronary microcirculatory dysfunction (8, 9). However, the additional procedure time, increased procedural costs, and the requirement for maximal hyperemia may hinder its use in clinical practice. This is particularly true in STEMI patients undergoing primary PCI, as it further increases patient risk. In recent years, coronary angiography-derived index of microcirculatory resistance (caIMR) has been extensively validated for accuracy and is widely used to assess CMD (10–12). Compared to traditional IMR, caIMR does not require pressure wires or adenosine, making it safer and more convenient. Previous studies have shown that CMD in STEMI patients significantly impacts prognosis (7, 13, 14). Notably, in one study, Thrombolysis in Myocardial Infarction (TIMI) was identified as an independent predictor of CI-AKI after PCI in STEMI patients (15). Additionally, nicorandil has been shown to effectively prevent CI-AKI, likely through its beneficial effects on microcirculation (16–18). To date, the relationship between IMR and CI-AKI remains unclear. This study aims to explore the relationship between caIMR and CI-AKI following primary PCI in STEMI patients.

## Methods

### Study population

This retrospective study included patients diagnosed with STEMI (19) between January 2021 and November 2024. The inclusion criteria were: (1) successful primary PCI performed within 12 h of symptom onset (TIMI = 3); (2) availability of complete clinical data. Patients were excluded if they met any of the following criteria: (1) pre-admission dialysis or chronic renal failure (eGFR <30 ml·min^−1^·1.73 m^−2^); (2) active inflammatory conditions (such as pulmonary infection, intestinal inflammation, or autoimmune diseases); (3) history of malignancy or hematologic disorders; (4) exposure to other radiographic contrast agents or nephrotoxic medications within 48 h before or 72 h after the procedure; (5) Poor CAG images or insufficient caIMR measurements. The study was approved by the Institutional Review Board (IRB) of the Affiliated Hospital of Xuzhou Medical University (No. XYFY2023-KL203-01). As a retrospective study posing no risk to patients, the requirement for written informed consent was waived in accordance with IRB guidelines. A total of 745 patients were enrolled in the study.

### Clinical data collection

Clinical data were obtained from patient records, including age, sex, body mass index (BMI), medical history, medications, left ventricular ejection fraction (LVEF), and PCI-related information. Pre-PCI serum creatinine (Scr) levels, as well as Scr measurements taken 48–72 h after contrast agent exposure, were documented. Contrast-induced acute kidney injury (CI-AKI) was defined as a Scr increase of at least 50% or 0.3 mg/dl within 48–72 h following contrast exposure (20). According to the relevant previous standards (21, 22), chronic kidney disease (CKD) and estimated glomerular filtration rate (eGFR) were calculated and defined. Additionally, peak levels of C-reactive protein (CRP), high-sensitivity troponin T (hs-TnT), and N-terminal pro B-type natriuretic peptide (NT-proBNP) during hospitalization were recorded. Medications prescribed included aspirin, P2Y12 inhibitors, β-blockers, statins, nitrates, angiotensin-converting enzyme inhibitors (ACEI) or angiotensin receptor blockers (ARB), and diuretics.

### Measurement of caIMR

**Figure 1:**
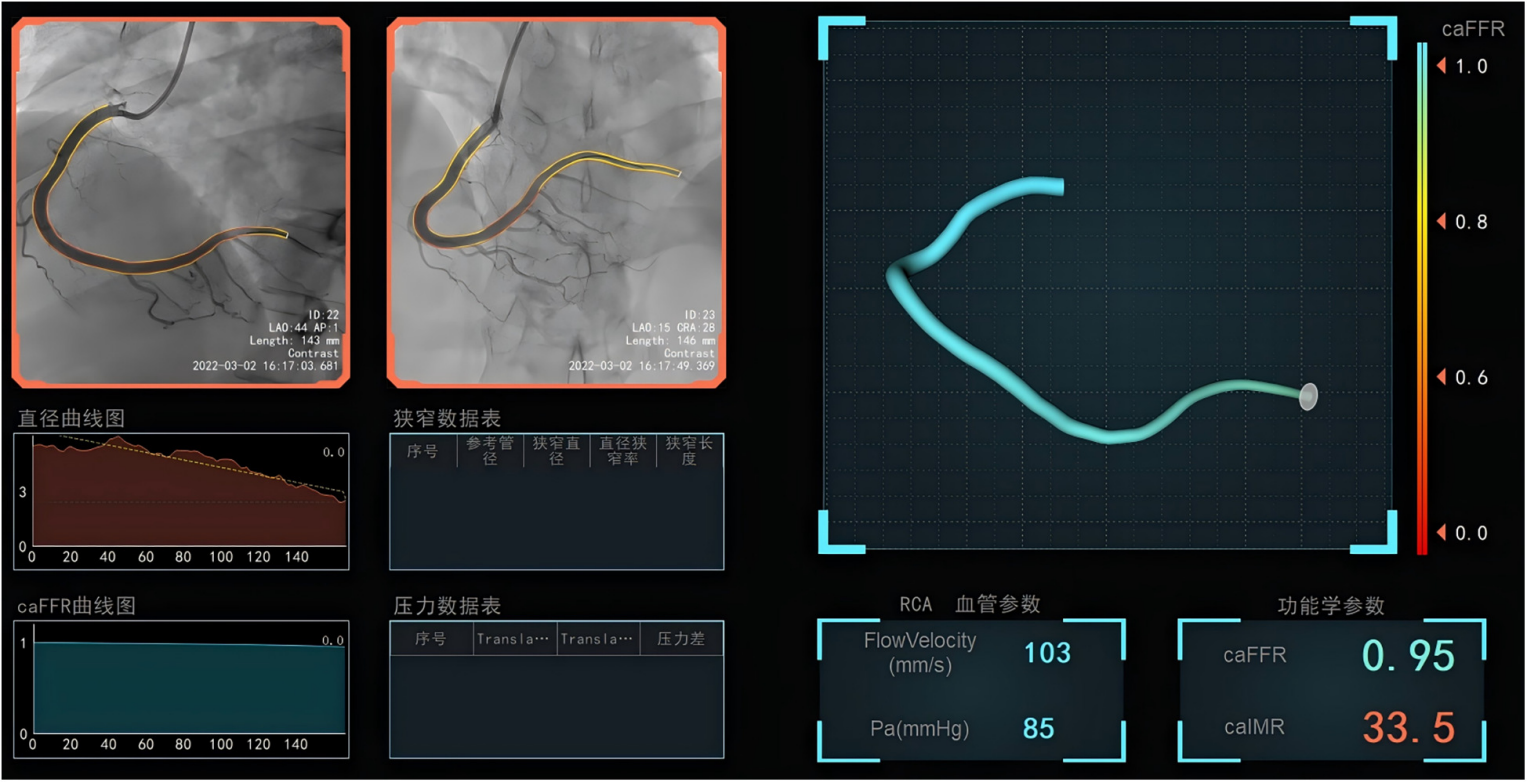
The measurement of coronary angiography-derived index of microcirculatory resistance in the infarct-related arteries after successful primary percutaneous coronary intervention.

The caIMR was assessed using commercial software (FlashAngio, Rainmed) (10). Two angiographic images of the target vessel, separated by at least 30°, were selected to create a three-dimensional reconstruction of the coronary artery. Invasive arterial pressure data from the guide catheter were obtained and entered into the FlashAngio console. The caIMR was calculated through a specialized hemodynamic method, as described by the formula: caIMR = Pd_hyp_ × (L/K·V_diastole_). Where Pd_hyp_ is the mean pressure at the distal site under maximal congestion. Pd_hyp_ was obtained based on the pressure recorded by CAG, and it is available for each patient. L is a constant representing the distance from the inlet to the distal site, V_diastole_ is the mean flow rate at the distal site during diastole, and K is another constant. Additionally, V_hyp_ = K·V represents the mean flow rate at the distal site under maximal congestion. PCI was performed by a team unaware of the study protocol, with hydration initiated within 12 h post-angiography according to the guideline (19). All angiograms were independently analyzed by core laboratory staff, who were blinded to the study data (Figure 1).

### Statistical analysis

Data were analyzed using SPSS (version 27.0, Chicago, USA) and R (version 4.3.1). The Kolmogorov–Smirnov test was applied to assess data normality. Continuous variables with a normal distribution were presented as mean ± standard deviation and compared using independent *t*-tests. Non-normally distributed variables were reported as median (interquartile range) and analyzed with the Mann–Whitney *U*-test. Categorical variables were summarized as counts and percentages and compared using the χ^2^ test. All the variables with a *p*-value less than 0.10 in the univariate analysis were included in the multivariate regression analysis using a stepwise forward method to identify independent risk factors for CI-AKI. To examine the dose-response relationship between caIMR and CI-AKI, restricted cubic splines (RCS) were employed. The predictive accuracy of the new and baseline models was assessed with receiver operating characteristic (ROC) curves, net reclassification index (NRI), and integrated discrimination improvement (IDI). A *P*-value of <0.05 was considered statistically significant.

## Results

### Patient characteristics

This study included 745 patients, of which 27.4% were female, with a mean age of 63.38 ± 13.10 years. The overall incidence of CI-AKI during hospitalization was 10.6% (79/745). Patients in the CI-AKI group were older, had higher levels of fasting blood glucose (FBG), caIMR, CRP, hs-TnT, and NT-proBNP, and a higher prevalence of diabetes and left anterior descending artery (LAD) involvement. Additionally, they had a lower left ventricular ejection fraction (LVEF) compared to those in the control group. All differences were statistically significant (*P* < 0.05) (Table 1).

**Table 1:** Patient characteristics.

Variable	Total (*n* = 745)	No CI-AKI (*n* = 666)	CI-AKI (*n* = 79)	*P*
Age, years	63.38 ± 13.10	63.09 ± 13.33	65.89 ± 10.65	0.034
Female, *n* (%)	204 (27.38)	179 (26.88)	25 (31.65)	0.369
Heart rate, bpm	80.20 ± 14.41	80.03 ± 14.47	81.61 ± 13.90	0.358
SBP, mmHg	127.47 ± 20.52	127.30 ± 20.52	128.86 ± 20.60	0.524
DBP, mmHg	79.09 ± 14.09	79.08 ± 14.11	79.15 ± 13.98	0.968
BMI, kg/m^2^	24.69 ± 3.91	24.61 ± 3.87	25.39 ± 4.20	0.091
Smoking, *n* (%)	345 (46.31)	312 (46.85)	33 (41.77)	0.392
Hypertension, *n* (%)	338 (45.37)	297 (44.59)	41 (51.90)	0.218
Diabetes, *n* (%)	187 (25.10)	159 (23.87)	28 (35.44)	0.025
CKD, *n* (%)	21 (2.82)	20 (3.00)	1 (1.27)	0.601
MI, *n* (%)	44 (5.91)	39 (5.86)	5 (6.33)	1.000
HGB, g/L	140.06 ± 16.85	140.13 ± 16.84	139.51 ± 17.07	0.756
Plt,10^9^/L	216.44 ± 59.68	217.22 ± 60.20	209.84 ± 54.92	0.298
Serum creatinine, μmol/L	67.27 ± 20.85	67.44 ± 20.97	65.81 ± 19.80	0.512
eGFR, ml/min/1.73 m^2^	102.07 ± 21.02	102.43 ± 20.92	99.07 ± 21.77	0.179
FBG, mmol/L	6.84 ± 2.84	6.69 ± 2.70	8.11 ± 3.62	0.001
Total cholesterol, mmol/L	4.31 ± 1.00	4.30 ± 1.01	4.34 ± 0.91	0.744
Triglycerides, mmol/L	1.50 ± 1.07	1.51 ± 1.10	1.37 ± 0.69	0.272
HDL-C, mmol/L	0.99 ± 0.24	0.98 ± 0.25	1.01 ± 0.15	0.211
LDL-C, mmol/L	2.77 ± 0.87	2.76 ± 0.88	2.85 ± 0.82	0.419
caIMR, U	23.46 ± 10.93	22.36 ± 9.96	32.69 ± 14.04	<0.001
Peak hs-CRP, mg/L	2.40 (0.60, 8.00)	2.30 (0.50, 7.77)	2.80 (1.49, 10.20)	0.008
Peak hs-TnT, ng/L	478.8 (95.0, 1,781.0)	452.2 (77.4, 1,876.3)	714.8 (167.7, 1,512.5)	0.091
Peak NT-proBNP, pg/ml	1,287.0 (522.0, 3,101.0)	1,159.5 (464.3, 2,882.4)	2,806.0 (1,348.5, 4,190.0)	<0.001
LVEF, %	51.96 ± 7.00	52.43 ± 6.83	47.99 ± 7.21	<0.001
IABP, *n* (%)	23 (3.09)	18 (2.70)	5 (6.33)	0.156
Killip class, *n* (%)				
I	634 (85.10)	572 (85.89)	62 (78.48)	0.180
II	36 (4.83)	32 (4.80)	4 (5.06)	
III	3 (0.40)	3 (0.45)	0 (0.00)	
IV	72 (9.66)	59 (8.86)	13 (16.46)	
Infarct-related arteries, *n* (%)				
LAD, *n* (%)	364 (48.86)	317 (47.60)	47 (59.49)	0.046
LCX *n* (%)	77 (10.34)	69 (10.36)	8 (10.13)	0.949
RCA, *n* (%)	301 (40.40)	277 (41.59)	24 (30.38)	0.055
Left main, *n* (%)	3 (0.40)	3 (0.45)	0 (0.00)	1.000
Aspirin, *n* (%)	743 (99.73)	664 (99.70)	79 (100.00)	1.000
P2Y12, *n* (%)	744 (99.87)	665 (99.85)	79 (100.00)	1.000
Statins, *n* (%)	741 (99.46)	662 (99.40)	79 (100.00)	1.000
ACEI/ARB/Sac/Val, *n* (%)	354 (47.52)	313 (47.00)	41 (51.90)	0.409
β-blockers, *n* (%)	653 (87.65)	585 (87.84)	68 (86.08)	0.653
Nitrates, *n* (%)	297 (39.87)	269 (40.39)	28 (35.44)	0.396
Heparin, *n* (%)	619 (83.09)	555 (83.33)	64 (81.01)	0.603
Diuretics, *n* (%)	406 (54.50)	355 (53.30)	51 (64.56)	0.058

BMI, body mass index; IABP, intra-aortic balloon pump; LVEF, left ventricular ejection fraction; CKD, chronic kidney disease; SBP, systolic blood pressure; DBP, diastolic blood pressure; LAD, left anterior descending; LCX, left circumflex artery; RCA, right coronary artery; ACEI, angiotensin-converting-enzyme inhibitor; ARB, angiotensin II receptor blocker; HDL-C, high-density leptin cholesterol; LDL-C, low-density leptin cholesterol; hs-CRP, high sensitivity C-reactive protein; hs-TnT, high sensitivity troponin T; NT-proBNP, N-terminal pro-B-type natriuretic peptide; FBG, fasting blood glucose; MI, myocardial infarction; caIMR, coronary angiography-derived index of microcirculatory resistance; CI-AKI, contrast-induced acute kidney injury.

### Logistic regression analysis of Ci-AKI

**Figure 2:**
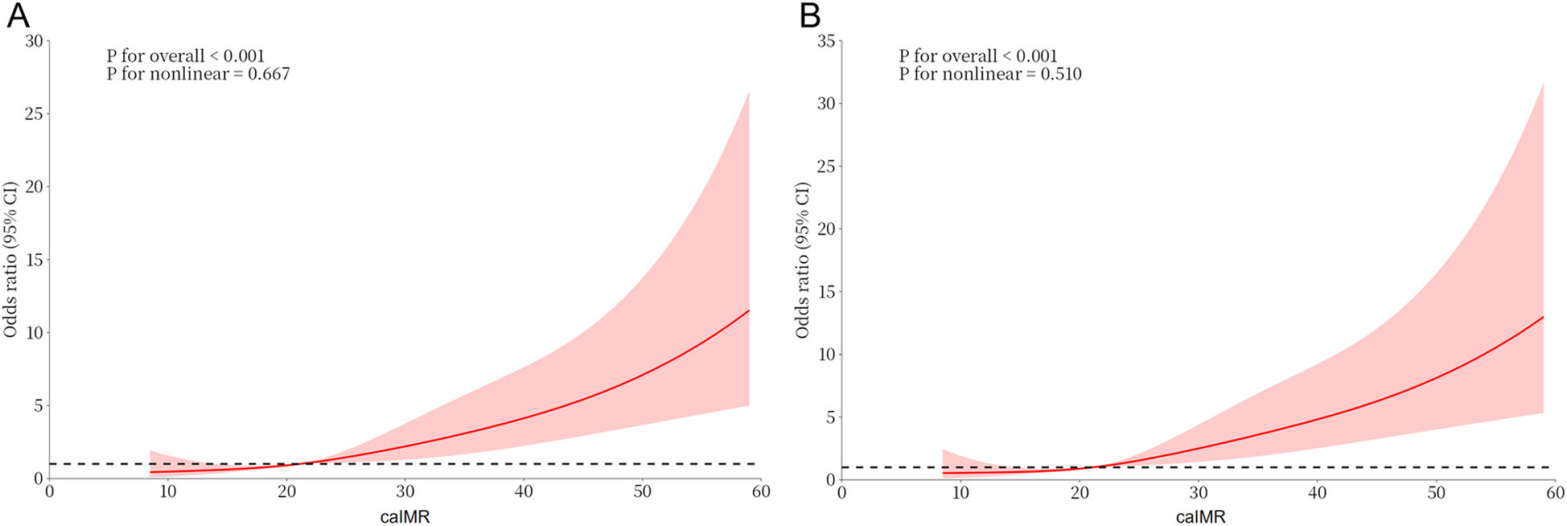
Dose-response relationship between caIMR and CI-AKI. **(A)** A unadjusted dose-response relationship between caIMR and CI-AKI; **(B)** an adjusted dose-response relationship between caIMR and CI-AKI. CI-AKI, contrast-induced acute kidney injury; caIMR, coronary angiography-derived index of microcirculatory resistance.

Univariate logistic regression revealed that caIMR (OR = 1.070, 95% CI: 1.050–1.090), FBG, NT-proBNP, diabetes, LAD, and LVEF were significantly associated with the development of CI-AKI during hospitalization (*P* < 0.05). When the caIMR increases by 10 U each time, the OR was 1.965 (95% CI: 1.628–2.371) (Supplementary Table S1). Multivariate logistic regression, which included all variables with *P* < 0.10 in univariate logistic regression, identified LVEF (OR = 0.931, 95% CI: 0.900–0.964), FBG (OR = 1.105, 95% CI: 1.019–1.198), NT-proBNP (OR = 1.401, 95% CI: 1.132–1.735), and caIMR (OR = 1.072, 95% CI: 1.051–1.094) as independent predictors of CI-AKI. When the caIMR increases by 10 U each time, the OR was 2.010 (95% CI: 1.646–2.454) (Table 2). RCS analysis indicated a linear dose-response relationship between caIMR and CI-AKI both before and after adjustments, suggesting that higher caIMR levels are associated with an increased risk of CI-AKI (Figure 2).

**Table 2:** Multivariate logistic regression analysis of CI-AKI.

Variable	Model 1	
	OR (95% CI)	*P*
Peak NT-proBNP, pg/ml	1.401 (1.132–1.735)	0.002
FBG, mmol/L	1.105 (1.019–1.198)	0.015
caIMR, U^a^	1.072 (1.051–1.094)	<0.001
caIMR, U (Increased each 10 U)^b^	2.010 (1.646–2.454)	<0.001
LVEF, %	0.931 (0.900–0.964)	<0.001

LVEF, left ventricular ejection fraction; FBG, fasting blood glucose; caIMR, coronary angiography-derived index of microcirculatory resistance; NT-proBNP, N-terminal pro-B-type natriuretic peptide; CI-AKI, contrast-induced acute kidney injury.

^a^ Adjusting age, body mass index, diabetes, high sensitivity troponin T, NT-proBNP, FBG, caIMR, LVEF, intra-aortic balloon pump, killip class, left anterior descending, and diuretics.

^b^ Adjusting age, body mass index, diabetes, high sensitivity troponin T, NT-proBNP, FBG, caIMR (increased each 10 U), LVEF, intra-aortic balloon pump, killip class, left anterior descending, and diuretics.

### ROC analysis of CI-AKI

**Figure 3:**
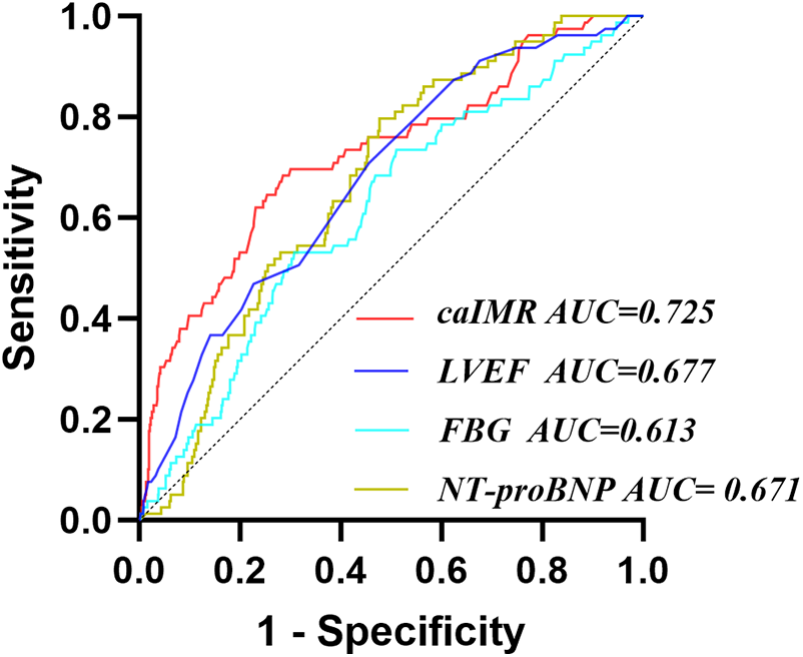
Receiver operating characteristic analysis (ROC) of caIMR for identifying CI-AKI. CI-AKI, contrast-induced acute kidney injury; caIMR, coronary angiography-derived index of microcirculatory resistance; LVEF, left ventricular ejection fraction; NT-proBNP, N-terminal pro-B-type natriuretic peptide; FBG, fasting blood glucose.

Receiver operating characteristic (ROC) analysis demonstrated that the areas under the curve (AUC) for LVEF, FBG, NT-proBNP, and caIMR in predicting CI-AKI were 0.677, 0.613, 0.671, and 0.725, respectively (*P* < 0.05). The optimal cutoff value for caIMR was 25.95 U, yielding a sensitivity of 68.4% and specificity of 71.5% (Table 3 and Figure 3).

**Table 3:** ROC analysis for CI-AKI.

Variable	AUC	95% CI	*P*	cut-off	Sensitivity	Specificity
LVEF, %	0.677	0.617–0.737	<0.001	51.5	0.709	0.544
NT-proBNP, pg/ml	0.671	0.617–0.725	<0.001	1,249.17	0.797	0.523
FBG, mmol/L	0.613	0.549–0.678	0.001	6.82	0.532	0.692
caIMR, U	0.725	0.662–0.789	<0.001	25.95	0.684	0.715

NT-proBNP, N-terminal pro-B-type natriuretic peptide; FBG, fasting blood glucose; caIMR, coronary angiography-derived index of microcirculatory resistance; LVEF, left ventricular ejection fraction.

### Comparison between baseline and new models

**Figure 4:**
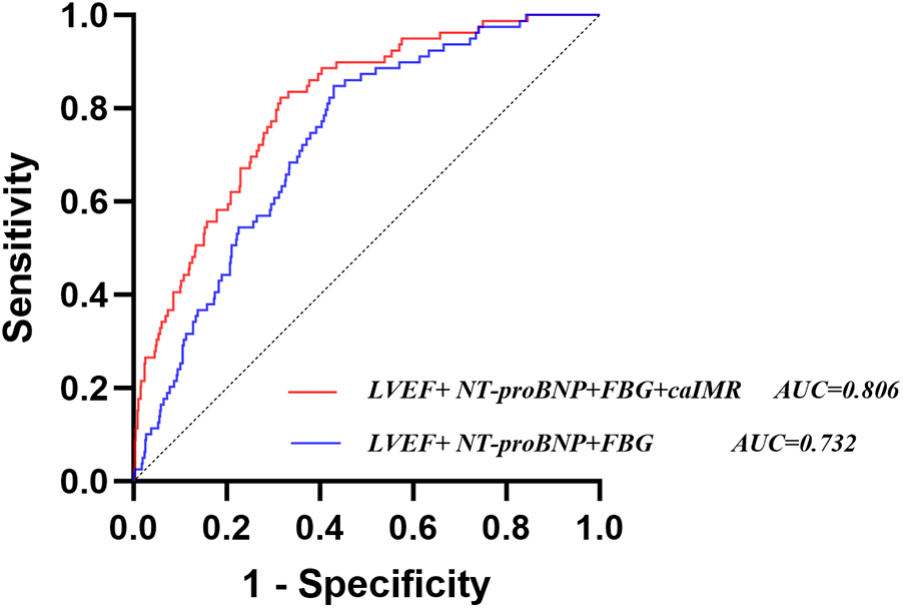
Receiver operating characteristic analysis (ROC) of models for identifying CI-AKI. CI-AKI, contrast-induced acute kidney injury; caIMR, coronary angiography-derived index of microcirculatory resistance; LVEF, left ventricular ejection fraction; NT-proBNP, N-terminal pro-B-type natriuretic peptide; FBG, fasting blood glucose.

Constructing a new model (LVEF, FBG, NT-proBNP, and caIMR) after integrating caIMR in a baseline model (LVEF, FBG, and NT-proBNP). ROC analysis showed that the baseline model had an AUC of 0.732 (95% CI: 0.682–0.783), with a sensitivity of 84.8% and specificity of 57.1%. The new model had an AUC of 0.806 (95% CI: 0.759–0.853), with a sensitivity of 82.3% and specificity of 68.5%. NRI and IDI for the new model were 0.721 (95% CI: 0.4968–0.9452), *P* < 0.001, and 0.102 (95% CI: 0.0626–0.1412), *P* < 0.001, respectively. These findings indicate that the new model significantly improves the risk model for CI-AKI in STEMI patients (Table 4, Figure 4 and Supplementary Table S2).

**Table 4:** Incremental value of caIMR for CI-AKI.

Variable	NRI		IDI	
	Estimate (95% CI)	*P*	Estimate (95% CI)	*P*
FBG + LVEF + NT-proBNP	Reference	–	Reference	–
FBG + LVEF + NT-proBNP + caIMR	0.721 (0.4968–0.9452)	<0.001	0.102 (0.0626–0.1412)	<0.001

NT-proBNP, N-terminal pro-B-type natriuretic peptide; FBG, fasting blood glucose; caIMR, coronary angiography-derived index of microcirculatory resistance; LVEF, left ventricular ejection fraction.

## Discussion

To our knowledge, this is the first study on the relationship between CMD and CI-AKI. The main findings of this study are as follows: first, elevated caIMR is an independent risk factor for the development of CI-AKI after PCI in STEMI patients; second, there is a linear dose-response relationship between caIMR and CI-AKI; and third, integrating caIMR significantly improves the risk model for CI-AKI.

CI-AKI is characterized by kidney dysfunction occurring within 48–72 h after the administration of contrast agents (23). Although recent evidence suggests that the risk of CI-AKI may have been overestimated, an important prerequisite is the potential exclusion of high-risk patients (24). Numerous studies have shown that CKD patients are less likely to undergo coronary angiography or PCI due to concerns about worsening kidney function (25, 26). In our study, 10.6% of patients still developed CI-AKI. Given the relationship between CI-AKI and poor outcomes (27, 28), and the limited pharmacological treatment options (29), identifying additional risk factors and optimizing risk stratification are essential.

Despite the successful restoration of epicardial coronary flow in STEMI patients via PCI, more than half of STEMI patients still experience myocardial reperfusion injury due to the presence of coronary microcirculatory dysfunction (CMD) (6, 7). In our study, there were 277 (37.2%) patients whose caIMR was greater than 25 U. The reason why CMD is at a relatively low level may be that all the patients included in our study have a TIMI flow grade of 3. Current clinical tools for assessing CMD include cardiac magnetic resonance imaging (CMR) and the index of microcirculatory resistance (IMR). While CMR is non-invasive, its use is limited by indications and high costs, meaning that not all STEMI patients undergo this examination (30). IMR, although the “gold standard” for evaluating microcirculatory dysfunction, requires a pressure-temperature sensing wire and induced maximal hyperemia, which limits its routine clinical application, especially in STEMI patients undergoing primary PCI (8, 9). Recently, caIMR, as an alternative to traditional IMR, has been widely used for prognosis stratification in STEMI patients (10–12). There is a significant correlation (R = 0.782, *P* < 0.001) and consistency (Bias = −0.398, SD of Bias = 11.96) between caIMR and traditional IMR (10). Given its simplicity and safety, caIMR is particularly beneficial in STEMI patients. In a previous study, Çınar et al. demonstrated that TIMI was independently associated with CI-AKI in STEMI patients undergoing primary PCI and provided better predictive value for CI-AKI than traditional risk factors (15). Nicorandil, a commonly used drug for improving microcirculatory dysfunction, has been shown in prior studies to effectively prevent CI-AKI, whether administered intravenously or orally (16–18). Consistent with these findings, our study identified elevated caIMR as an independent risk factor for the development of CI-AKI after PCI in STEMI patients, and we found a linear dose-response relationship between caIMR and CI-AKI. Given the established relationship between CMD and poor prognosis in STEMI patients, our findings appear to be well-founded. Previous studies have shown that CMD in STEMI patients is associated with larger infarct sizes, worse left ventricular remodeling, and intense inflammatory response, which play an important role in the mechanism of CI-AKI (3, 4, 7, 13, 14). Additionally, AKI is closely associated with endothelial dysfunction (31). It is known that endothelial dysfunction is also an important mechanism of CMD. Therefore, systemic endothelial dysfunction may be a potential mechanism underlying the relationship between caIMR and CI-AKI. In our study, ROC analysis showed that caIMR is a strong predictor of CI-AKI, outperforming FBG, LVEF, and NT-proBNP, aligning with the previous study (15). In previous studies, a threshold value of 25 U for either caIMR or traditional IMR has been proposed for diagnosing microcirculatory dysfunction (8, 9, 32, 33). Interestingly, in our study, we found that a caIMR value of 25.95 U was the cutoff for predicting CI-AKI in STEMI patients after PCI. To our knowledge, this is the first study to establish a cutoff for caIMR in relation to CI-AKI, potentially providing additional insights for IMR-based prognosis assessment in STEMI patients. Differently, in another previous study on STEMI patients, the cutoff value of the angiography-derived IMR was higher than that in our study. This may be attributed to the differences in the measurement software for IMR and the end-point events (34). Our study also demonstrates that caIMR can effectively enhance risk models for CI-AKI. Given its simplicity and safety, caIMR represents a valuable tool for optimizing risk stratification for CI-AKI after PCI in STEMI patients. These findings suggest that patients with elevated caIMR may require more clinical attention and intervention to reduce the risk of CI-AKI.

There are several limitations to this study. First, given its retrospective and observational design, the current study remains inherently descriptive, hypothesis-generating, and speculative in nature. While our study provides a compelling association between CMD and CI-AKI, the capacity for causal inference remains limited. The observed elevation in caIMR in these patients may not solely reflect isolated microvascular dysfunction but rather a broader picture of myocardial damage, impaired reperfusion, and a generally more adverse in-hospital clinical course. Consequently, caIMR might function as a surrogate marker of overall disease severity rather than a direct mechanistic contributor to CI-AKI. Second, the sample size is relatively small, and some conclusions may need to be validated in larger cohorts. Third, this study specifically involves STEMI patients who underwent successful primary PCI, meaning the findings may not be applicable to other patient populations. Fourth, in our study, caIMR was evaluated after the completion of PCI, which made strategies to reduce the use of contrast agents no longer feasible. This limits the clinical utility of caIMR as a tool for early risk stratification or surgical planning. For STEMI patients undergoing primary PCI, the practical measurement of caIMR at an earlier stage may still be a significant challenge. In addition, the implementation of postoperative renal protection strategies mainly based on saline hydration may precisely be restricted in the subgroup of patients identified as high-risk, who have poorer cardiac function. These factors highlight the importance of considering caIMR in a way that can effectively provide a basis for personalized treatment decisions. Fifth, given that the application of functional coronary angiography remains limited in clinical practice, it may still be too early to incorporate caIMR into the prediction model for CI-AKI. Finally, CI-AKI is a surrogate marker for adverse outcomes, but whether the elevated caIMR is associated with persistent renal dysfunction or mortality may require more follow-up in the future to determine.

## Conclusion

Elevated caIMR is an independent risk factor for the development of CI-AKI after PCI in STEMI patients. Integrating caIMR significantly improves the risk model for CI-AKI.

## Data Availability

The raw data supporting the conclusions of this article will be made available by the authors, without undue reservation.
