# Supplementary material for: Coronary angiography–derived index of microcirculatory resistance associated with contrast-induced acute kidney injury in patients with STEMI
Source: Front Cardiovasc Med. 2025 May 1;12:1541208. doi: 10.3389/fcvm.2025.1541208 (PMC12078307; doi:10.3389/fcvm.2025.1541208)
Supplement: Supplementary file 1 [file Table1.docx]

**Supplementary Table 1. Univariate Logistic Regression Analysis of CI-AKI**

|  | OR (95%CI) | *P* |
| --- | --- | --- |
| Age, years | 1.017 (0.998 ~ 1.036) | 0.073 |
| Female, n (%) | 1.260 (0.761 ~ 2.085) | 0.370 |
| Heart rate, bpm | 1.008 (0.992 ~ 1.024) | 0.358 |
| SBP, mmHg | 1.004 (0.992 ~ 1.015) | 0.524 |
| DBP, mmHg | 1.000 (0.984 ~ 1.017) | 0.968 |
| BMI, kg/m^2^ | 1.051 (0.992 ~ 1.114) | 0.092 |
| Smoking, n (%) | 0.814 (0.508 ~ 1.305) | 0.393 |
| Hypertension, n (%) | 1.341 (0.840 ~ 2.138) | 0.219 |
| Diabetes, n (%) | 1.751 (1.068 ~ 2.870) | 0.026 |
| CKD, n (%) | 0.414 (0.055 ~ 3.128) | 0.393 |
| MI, n (%) | 1.086 (0.415 ~ 2.842) | 0.866 |
| HGB, g/L | 0.998 (0.984 ~ 1.012) | 0.755 |
| Plt, 10^9/L | 0.998 (0.994 ~ 1.002) | 0.298 |
| Peak hs-CRP, mg/L | 1.005 (0.997 ~ 1.012) | 0.215 |
| Serum creatinine, μmol/L | 0.996 (0.984 ~ 1.008) | 0.511 |
| eGFR, mL/min/1.73 m^2^ | 0.993 (0.982 ~ 1.003) | 0.180 |
| Peak hs-TnT, ng/L | 1.128 (0.991 ~ 1.284) | 0.068 |
| Peak NT-proBNP, pg/mL | 1.520 (1.257 ~ 1.838) | <0.001 |
| FBG, mmol/L | 1.143 (1.071 ~ 1.221) | <0.001 |
| Total cholesterol, mmol/L | 1.039 (0.825 ~ 1.310) | 0.744 |
| Triglycerides, mmol/L | 0.853 (0.643 ~ 1.132) | 0.271 |
| HDL-C, mmol/L | 1.514 (0.586 ~ 3.913) | 0.392 |
| LDL-C, mmol/L | 1.114 (0.858 ~ 1.448) | 0.418 |
| caIMR, U | 1.070 (1.050 ~ 1.090) | <0.001 |
| caIMR, U (Increased each 10 U) | 1.965 (1.628 ~ 2.371) | <0.001 |
| LVEF, % | 0.920 (0.891 ~ 0.949) | <0.001 |
| IABP, n (%) | 2.432 (0.877 ~ 6.743) | 0.087 |
| Killip class >1, n (%) | 1.668 (0.935 ~ 2.978) | 0.083 |
| IRA-LAD, n(%) | 1.617 (1.006 ~ 2.598) | 0.047 |
| IRA-LCX n(%) | 0.975 (0.450 ~ 2.110) | 0.949 |
| IRA-RCA, n(%) | 0.613 (0.370 ~ 1.014) | 0.057 |
| ACEI/ARB/Sac/Val, n(%) | 1.217 (0.763 ~ 1.941) | 0.410 |
| β-blockers, n(%) | 0.856 (0.435 ~ 1.686) | 0.653 |
| Nitrates, n(%) | 0.810 (0.498 ~ 1.318) | 0.396 |
| Heparin, n(%) | 0.853 (0.469 ~ 1.552) | 0.603 |
| Diuretics, n(%) | 1.596 (0.982 ~ 2.593) | 0.059 |

BMI = body Mass Index; IABP = intra-aortic balloon pump; LVEF = left ventricular ejection fraction; CKD = chronic kidney disease; SBP = systolic blood pressure; DBP = diastolic blood pressure; LAD = left anterior descending; LCX = left circumflex artery; RCA = right coronary artery; ACEI = angiotensin-converting-enzyme inhibitor; ARB = angiotensin II receptor blocker; HDL-C = high-density leptin cholesterol; LDL-C = low-density leptin cholesterol; hs-CRP = high sensitivity C-reactive protein; hs-TnT = high sensitivity troponin T; NT-proBNP = N-terminal pro-B-type natriuretic peptide; FBG = fasting blood glucose; MI = myocardial infarction; caIMR = coronary angiography-derived index of microcirculatory resistance; CI-AKI = contrast-induced acute kidney injury.
