# Supplementary material for: Coronary angiography–derived index of microcirculatory resistance associated with contrast-induced acute kidney injury in patients with STEMI
Source: Front Cardiovasc Med. 2025 May 1;12:1541208. doi: 10.3389/fcvm.2025.1541208 (PMC12078307; doi:10.3389/fcvm.2025.1541208)
Supplement: Supplementary file 2 [file Table2.docx]

**Supplementary Table 2. ROC Analysis of Models for CI-AKI**

|  | AUC | 95%CI | *P* | Sensitivity | Specificity |
| --- | --- | --- | --- | --- | --- |
| LVEF+FBG+NT-proBNP | 0.732 | 0.682~0.783 | <0.001 | 0.848 | 0.571 |
| LVEF+FBG+NT-proBNP+caIMR | 0.806 | 0.759~0.853 | <0.001 | 0.823 | 0.685 |

NT-proBNP = N-terminal pro-B-type natriuretic peptide; FBG = fasting blood glucose; caIMR = coronary angiography-derived index of microcirculatory resistance; LVEF = left ventricular ejection fraction.
